# 68Ga-labeled PSMA-11 (68Ga-isoPROtrace-11) synthesized with ready to use kit: normal biodistribution and uptake characteristics of tumour lesions

**DOI:** 10.1038/s41598-020-60099-y

**Published:** 2020-02-20

**Authors:** Marina Muchnik Kurash, Ronit Gill, Maria Khairulin, Hanan Harbosh, Zohar Keidar

**Affiliations:** 1 Department of Nuclear Medicine, Rambam Health Care Campus, Haifa, Israel; 2 The Ruth and Bruce Rappaport Faculty of Medicine, Technion – Israel Institute of Technology, Haifa, Israel

**Keywords:** Diseases, Medical research, Molecular medicine, Oncology, Urology

## Abstract

68Ga-PSMA-11, the radiotracer of choice for imaging of prostate cancer (PCa), may be produced with several radiolabeling techniques. Current study aimed to analyze various imaging parameters of the cold kit methodology produced 68Ga-PSMA-11 (68Ga-isoPROtrace-11) and to compare the results to available data in literature. Eighty 68Ga-PSMA-11 positron emission tomography/computed tomography (PET/CT) scans were evaluated. 68Ga-isoPROtrace-11 for all the studies was produced by the room temperature cold kit methodology using a lyophilized ready-to-use vial. Normal biodistribution of the tracer was recorded by measuring mean standardized uptake value (SUVmean) and compared to the available published data. Pathological tracer uptake was measured using SUVmax in prostate gland (48 patients), lymph nodes (22 patients), bones (20 patients) and soft tissues (6 patients). Average tumour-to-background and tumour-to-liver contrast ratios were calculated. The data of 80 68Ga-PSMA-11 PET/CT scans were analyzed. Radiochemical purity of the tracer was 91% or more. The highest normal tissue uptake value of 68Ga-isoPROtrace-11 was found in the kidneys (average SUVmean 41.7), followed by the parotid (average SUVmean 14.5) and submandibular glands (average SUVmean 13.02). Normal prostate tissue showed low tracer uptake (average SUVmean 2.4). The biodistribution of 68Ga-isoPROtrace-11 in normal tissues was found to be similar to other published results. Pathological uptake (average SUVmax ± standard deviation) in prostate gland was 11.3 ± 7.5, in lymph node metastases 14.6 ± 13.7, in bones 15.9 ± 15.9 and 24.2 ± 16.4 in soft tissues. Average tumour uptake of 68Ga-isoPROtrace-11 in prostate was 11.3, in lymph node metastases 14.6, in bone metastases 15.9 and in soft tissue metastases 24.2. Average tumour-to-liver and tumour-to-mediastinal blood pool ratios were 2.7 and 13.54 respectively. This study presents biodistribution data of 68Ga-isoPROtrace-11 in a large PCa patient subset, showing clinical applicability of the tracer. Using cold kit technology may enable a high quality and easy labeling process.

## Introduction

Prostate cancer (PCa) is the second most frequent malignancy (after lung cancer) in men worldwide^1^. The most common diagnostic procedures for diagnosing PCa for over 30 years have been physical examination (i.e., the digital rectal examination), serum prostate specific antigen (PSA) levels, imaging tests and biopsy, either trans-rectal, trans-perineal, blind or image-directed.

According to guidelines, imaging should be performed for the detection and characterization of disease in order to select treatment or guide patient management. Different anatomic and functional imaging tests are recommended depending on the patient’s risk group^2^. One of the major developments, occurred in molecular imaging in recent years, involves detection of prostate-specific membrane antigen (PSMA) with positron emission tomography/computed tomography imaging technique (PET/CT).

PSMA is a cell surface transmembrane protein that is over-expressed in most PCa cells^3–6^. Radiolabeled PSMA ligand Glu-NH-CO–NH-Lys-(Ahx)-[68Ga (HBED-CC)], also known as 68Ga-PSMA-11, is currently the most popular radiotracer used for PET imaging of prostate cancer. It offers attractive imaging characteristics including favorable tumour-to-background ratio and shows correlation between the tumour-related tracer uptake intensity and PSA levels and Gleason scores^7,8^.

68Ga-PSMA-11 PET/CT is widely used in several clinical indications including staging of intermediate- to high-risk PCa patients or restaging in patients with biochemical recurrence, showing superiority to choline PET/CT due to its higher accuracy^9–13^.

The availability of 68Ge/68Ga generators and PSMA-11 peptide from commercial sources enables in-house production of the radiopharmaceutical using a manual or, more common, automated synthesis module (synthesizer). These methods are time consuming, require highly qualified personnel and expensive equipment, in addition to high grade purity of consumables and reagents.

Availability of a simple 1-step labeling process would lead to benefits in logistics and clinical use of the tracer. A method of using a ready-to-use vial is a new trend emerging in the last years. It may enable a very easy, short and efficient radiolabeling process. Several cold kits for 68Ga-PSMA production have been recently introduced in clinical studies, including ANMI and THP that have demonstrated to be safe and acceptable for clinical imaging with no significant differences comparing to 68Ga-PSMA ligands produced via synthesis modules^14,15^.

The technique of using a lyophilized cold kit (isoPROtrace-11) was applied for the production of 68Ga-PSMA-11 used for PET/CT scans and serves as the major subject of the current study. In this study, we aimed to analyze the a variety of imaging parameters of 68-Ga-PSMA-11 produced with a room temperature cold kit methodology, to present a detailed biodistribution map in healthy tissues and to compare these results to available published data, to show uptake characteristics in prostate cancer lesions.

## Materials and Methods

### Patients

Eighty consecutive patients with prostate cancer who underwent 68Ga-PSMA PET/CT between February 2019 and April 2019 at the department of Nuclear Medicine were retrospectively evaluated. All patients referred to 68Ga-PSMA PET/CT within this period were included in the study. Age, Body Mass Index (BMI), clinical indication for the scan, recent prostate-specific antigen (PSA) levels and Gleason score were extracted from the patients’ records (Tables 1, 2). This retrospective study was approved by the Ethics Committee of the Rambam Health Care Campus (permit 0078-19-RMB) and all reported investigations were conducted in accordance with the national regulations. Patient informed consent has been waived.

**Table 1:** Baseline clinico-pathologic characteristics of 80 patients.

	Mean ± SD	Median
Age (y)	72.7 (8.8)	72
Weight (kg)	80.4 (13.6)	78
BMI (kg/m²)	27.9 (5.7)	27
Injected activity (MBq)	4.8 (0.6)	4.8
Uptake time (min)	56 (5.9)	55
Recent PSA (ng/mL)	9.5 (18.1)	2.4
Total Gleason score	7.3 (1.2)	7

**Table 2:** Clinical indications for the scans in 80 patients.

Clinical indication	Number of patients	Percentage
Primary staging or restaging of known recurrence	26	32.5
Biochemical failure	38	47.5
Assessment response to therapy	14	17.5
Follow-up	2	2.5

### Radiopharmaceutical production

The initial material for labeling was obtained from a 68Ge/68Ga-generator (IGG- 100, Art. No. 3131-0900) supplied by Eckert & Zeigler (EZAG, Berlin, Germany). The generator is a closed system consisting of a borosilicate glass column containing a titanium dioxide bed on which 68Ge is absorbed. 68Ga is continuously produced by decay of its radioactive parent and is eluted with 0.1 M HCI. The labeling process was carried out under good manufacturing practice conditions. All involved chemicals were of analytical grade and were used without additional purification. For radiolabeling with the lyophilized cold kit isoPROtrace-11, 2.5 mL 0.1 M HCl of the middle Ga-68 elution fraction were added to the kit through a 0.2 µm PVDF membrane syringe filter (Merck, Darmstadt, Germany) connected to the outlet of the generator via tubing. Following the addition of gallium-68, the vial was shook thoroughly for dissolving its’ contents and incubated for 5 minutes at room temperature. The amount of PSMA-11 in the kit was 10 micrograms. Radiochemical purity was assessed with the thin layer chromatography (TLC). Stationary phase used was TLC silica gel 60 (Merck, Darmstadt, Germany) with two mobile phases. The first is sodium citrate 0.1 M in water (Merck, Darmstadt, Germany) used to measure free gallium-68 as the first impurity presenting a retardation factor of 0.1–0.2, while the second mobile phase was 77 g/L solution of ammonium acetate in water/Methanol 50:50 V/V (Merck, Darmstadt, Germany) used to isolate the impurity of gallium-68 species (such as 68-Ga colloids) which presented by a retardation factor of 0.0–0.1. The percentage of radiochemical purity was calculated according to the following formula: 100% − (free gallium-68)% − (gallium-68 species)% and it was set to be not less than 91%.

### Patient preparation

After the preparation and quality control of the radiotracer, all patients received intravenously an average dose of 177.6 MBq (range 122.1–222 MBq) of 68Ga-PSMA-11. Following the injection, an oral contrast up to 1 liter was given for a continuous intake within 1 hour for optimal abdominopelvic imaging (Telebrix or E-Z-CAT, depending on known allergy to iodine). Patients were asked to void before imaging to reduce bladder activity and whole-body images were acquired 56 min in average (55 min median, range 45–71 min) post-injection of radiotracer. 54/80 (68%) patients received intravenous CT contrast enhancement, allowing performance of diagnostic CT as a part of PET/CT study in this group of patients. Criteria for CT contrast injection were glomerular filtration rate above 60 mL/min and no contraindications for the injection (known allergy to iodine, asthma, documented anaphylactic reaction, etc).

### Imaging protocol

All patients underwent PET/CT scans following standard protocol of the department with region of interest from skull base to upper thigh with six to seven bed positions (3 min/bed) on a Discovery 690 (GE Healthcare) PET/CT scan.

For those patients, who received intravenous contrast, the CT scans were of diagnostic quality.

### Image analysis

68Ga-PSMA PET/CT images were interpreted individually on a GE Xeleris workstation by two nuclear medicine physicians who were blinded to patients’ history and clinical information. Normal biodistribution of the tracer was analyzed measuring standardized uptake value (SUV) mean in healthy tissues, lowering maximal intensity of PET image to define the hottest area. Region of interest (ROI) of 1.5–2 cm (depending on organ size) was used when measuring SUVmean in normal tissues and was set manually for each patient. Physiological uptake was analyzed in the following tissues: brain, lacrimal glands, parotid glands, submandibular glands, mediastinal blood pool, liver, spleen, bowel, kidneys, pancreas and normal prostate tissue.

Pathological PSMA uptake was measured using SUVmax and defined as focal tracer uptake greater than normal or physiological local background activity and was measured with a standard automatically set ROI of 1.0 cm. It was analyzed in the prostate gland when pathological uptake was suspected, including areas of direct invasion into the adjacent organs, in lymph node metastases, soft tissue and bone metastases. If the findings were equivocal, the final decision was reached by consensus.

Tumour-to-background ratio was analyzed by dividing tumour uptake SUV measurements to the liver and mediastinal blood pool activities. Two parameters were obtained: average tumour-to-background ratio (using mediastinal blood pool as a background) and average tumour-to-liver contrast ratio.

## Results

The data of 80 prostate cancer patients who underwent 68Ga-PSMA PET/CT were analyzed. Patients’ age ranged from 56 to 89 (average 72), Gleason scores ranged from 5 to 10 with score of 7 being the most prevalent value 30/80 (38%). PSA level at the time of PET/CT study ranged between 0.01 to 132 ng/ml (average 9.5 ng/ml). Indications for PET/CT study included primary staging or restaging of known recurrence (26 patients, 32.5%), biochemical failure (38 patients, 47.5%), assessment response to therapy (14 patients 17.5%) and follow-up study in 2 patients. 68Ga-isoPROtrace-11 for all PET/CT scans was produced by the room temperature cold kit methodology using a lyophilized ready-to-use vial. During the production radiochemical purity was assessed with the TLC. The percentage of radiochemical purity was set to be 91% or more.

### Biodistribution of 68Ga-isoPROtrace-11in normal tissues, measured in SUVmean (average ± standard deviation)

**Figure 1:**
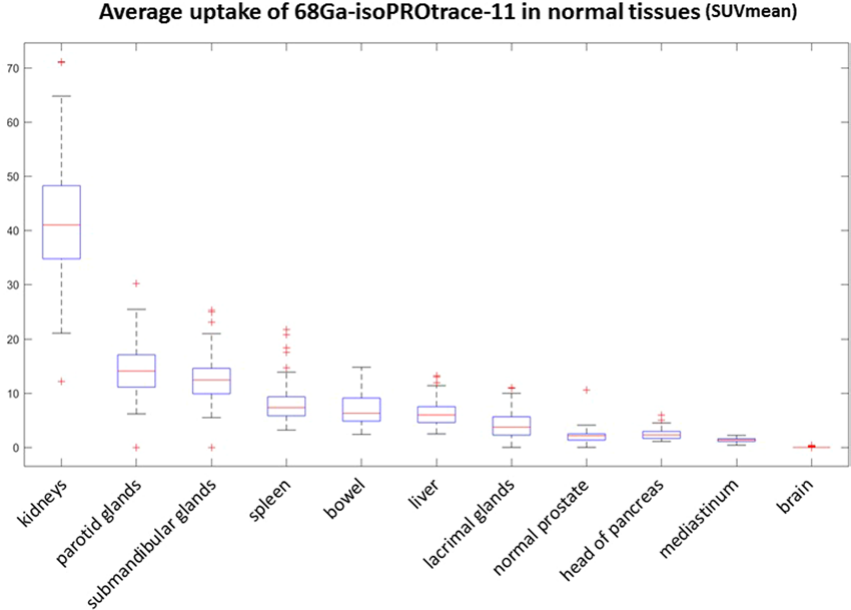
68Ga-isoPROtrace-11 uptake in normal tissues. Physiological uptake measured in SUVmean (range and average), shown from highest to lowest in the following organs: kidneys, parotid glands, submandibular glands, spleen, bowel, liver, lacrimal glands, normal prostate tissue, head of pancreas, mediastinal blood pool and brain.

**Figure 2:**
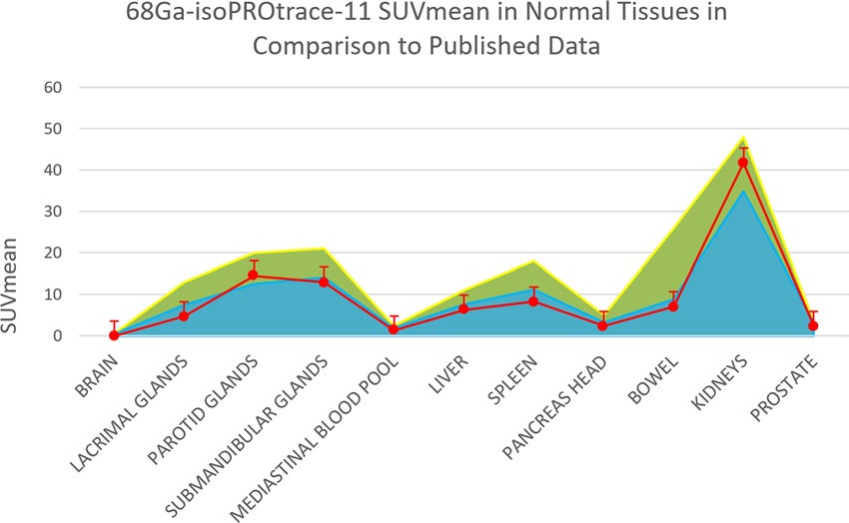
68Ga-isoPROtrace-11 SUVmean in normal tissues in comparison to published data: Physiological uptake measurements (SUVmean), obtained in the current study (red line) in comparison to two recently published references with uptake measurements in the same tissues (green with yellow boarder (14) and blue with dark blue boarder (15)).

Highest uptake of the tracer was found in the kidneys (41.7 ± 11.5), followed by the parotid (14.5 ± 4.6) and submandibular glands (13 ± 4.3), spleen (8.2 ± 3.7), bowel (7.1 ± 3) and liver (6.3 ± 2.3). Bowel uptake was found to be heterogeneous through its different parts with no specific prominent area of PSMA activity. Relatively low PSMA uptake was seen in the lacrimal glands (4.8 ± 2.4), head of pancreas (2.4 ± 0.9) and mediastinal blood pool (1.3 ± 0.4). Normal prostate tissue showed also low tracer uptake (2.4 ± 1.2). Brain tissue showed almost no uptake, with an average SUVmean of 0.03 (Fig. 1). The biodistribution of 68Ga-isoPROtrace-11 in normal tissues, measured in SUVmean, was found to be similar the most recently published results based on 68Ga-PSMA produced using the standard available methods^16,17^ (Fig. 2).

### Tumour uptake of 68Ga-isoPROtrace-11, measured in SUVmax (average ± standard deviation)

**Figure 3:**
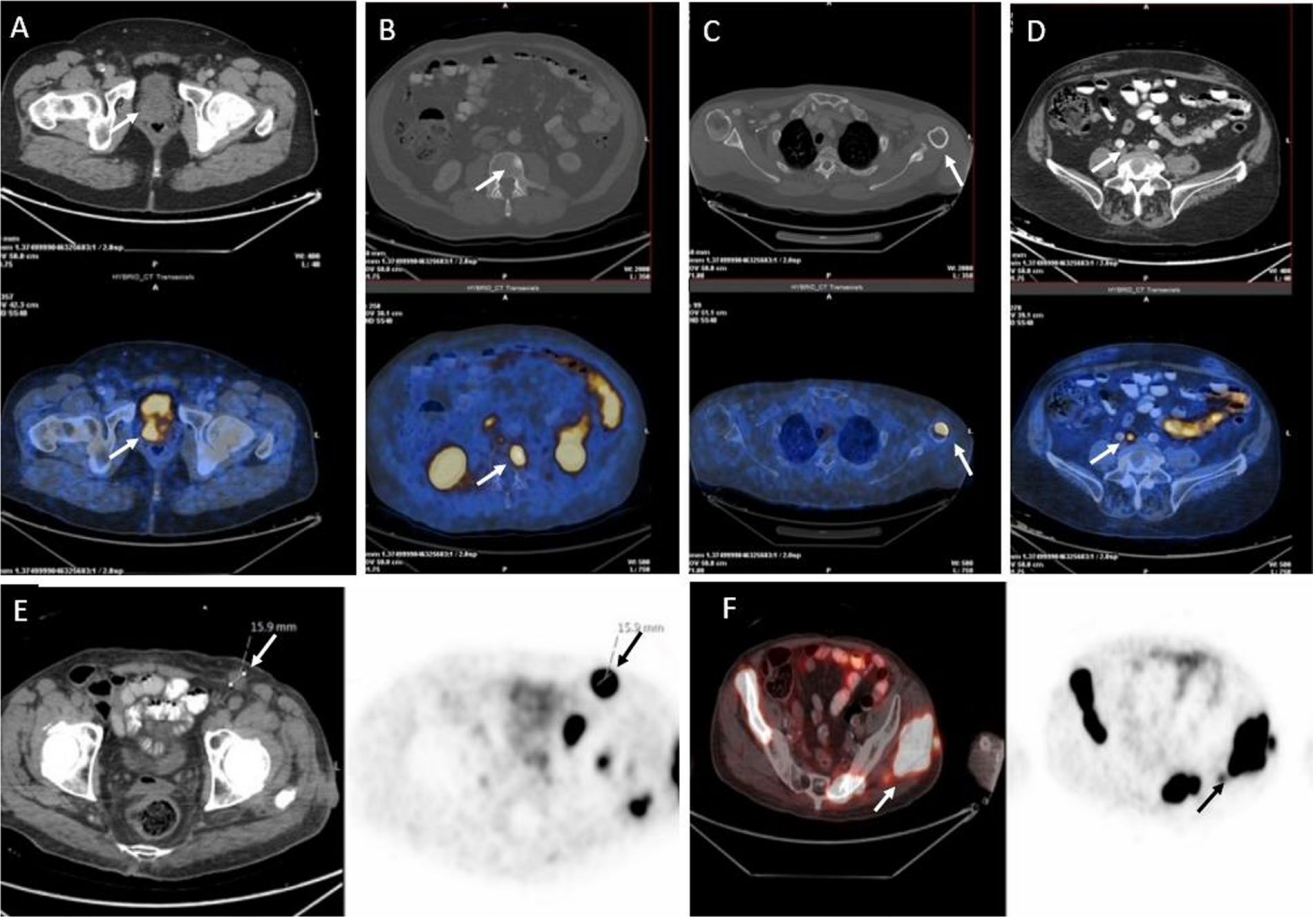
Examples of different sites showing pathological 68Ga-isoPROtrace-11 uptake: (**A**) Prostate tumour, (**B**) Bone metastasis with a CT finding, (**C**) Bone metastasis with no finding on CT, (**D**) Small (up to 15 mm) lymph node metastasis, (**E**) Enlarged (more than 15 mm) lymph node metastasis, (**F**) Soft tissue metastasis (see arrows).

Foci of 68Ga-isoPROtrace-11 uptake in sites considered to be malignant were demonstrated in 61 patients (76%) and were localized to the prostate gland, lymph nodes, bone and soft tissue (Fig. 3). Pathological prostate gland involvement was shown in 48 patients (60%) with SUVmax ranging from 3.5 to 40.6 (average SUVmax of 11.3 ± 7.5). Lymph node (LN) metastases were found in 22 patients (28%) with SUVmax varied from 3.7 to 59.3, showing average SUVmax of 14.6 ± 13.7. In total 69 LN were analyzed. Median number of nodal metastases with LN uptake was 2.5, ranging from 1 to uncountable (more than 20).

In addition, the CT parameters of suspected LN were analyzed. All LN metastases were measured in a short axis, transaxial view. A size of 15 mm was determined as a threshold, separating small from enlarged LN.

Three groups were identified according to LN size: Group A (small LN, in 19 patients), in total 57 pathological lymph nodes were measured, having an average size of 8 ± 3 mm. Average SUVmax in this group was 13.2.

Group B (Enlarged LN, one patient) showed pathological uptake of SUVmax 4.7 in one enlarged lymph node of 22 mm in diameter.

Group C (Both small and enlarged LN, 2 patients) had pathological uptake in 11 LN (9 small and 2 enlarged LN), having an average size of 10 ± 4 mm. The average SUVmax in this group of patients was 24.

Local metastases were counted and identified as invasion of the seminal vesicles or extra prostatic invasion of adjacent structures such as external sphincter, rectum, bladder, levator muscles and/or pelvic wall. Pelvic LN metastases up to the level of common iliac vessels were considered as local metastases as well. Nineteen patients (24%) had local metastases, but only one patient had a spread of more than 20 LN.

Metastatic bone disease was demonstrated in 20 patients (25%) ranging from one foci of pathological uptake to uncountable (more than 20) with a median of 4 and SUVmax ranging from 4.3 to 77 (average SUVmax of 15.9 ± 15.9). All these patients were divided into 2 groups depending on the number of bone metastases with 13 patients showing less than 20 sites and 5 more than 20 bone metastases. Two patients showed unclear single uptake in the skeleton and a diagnosis of benign finding was suggested (in one patient a fracture, and in another- artifact). In addition, all 20 patients with pathological bone uptake were divided into 2 groups based on the presence (Group D) or absence (Group E) of CT findings. The majority of the patients (18 of 20, Group D) showed pathological radiotracer uptake correlated to a suspicious CT finding (average SUVmax 13.5). Only two patients had pathological uptake without a CT finding showing SUVmax of 4.3 and 77 respectively. Soft tissue distant metastases were identified in 6 patients (8%). Two of these patients showed a widespread metastatic disease with more than 20 pathological foci. Average SUVmax in this group of findings was 24.2 ± 16.4. The distribution of 68Ga-isoPROtrace-11 in all pathological findings is summarized in Table 3.

**Table 3:** Pathological findings distribution of 68Ga-isoPROtrace-11 PET/CT in 80 patients.

	Total N of patients (%)	SUVmax range (average)	All patients, divided into groups based on CT findings		
Prostate tumour	48 (60%)	40.6–3.5 (11.3)	Not relevant		
Lymph node metastases	22 (28%)	3.7–59.3 (14.6)	Group A: ^a^19 patients 57 LN average SUVmax 13.2	Group B: ^b^1 patient 1 LN SUVmax 4.7	Group C: ^c^2 patients 11 LN average SUVmax 24
Bone metastases	20 (25%)	4.3–77 (15.9)	Group D: ^d^18 patients average SUVmax 13.5	Group E: ^e^2 patients average SUVmax 40.6	
Soft tissue distant metastases	6 (8%)	11–53.4 (24.2)	Not relevant		

^a^Group A: Small LN (N = 57, 8 ± 3 mm in diameter).

^b^Group B: One enlarged LN (22 mm in diameter).

^c^Group C: Both small and enlarged LN (N = 11, 10 ± 4 mm in diameter).

^d^Group D: Tracer uptake with a correspondent CT finding.

^e^Group E: Tracer uptake with no correspondent finding on CT.

**Figure 4:**
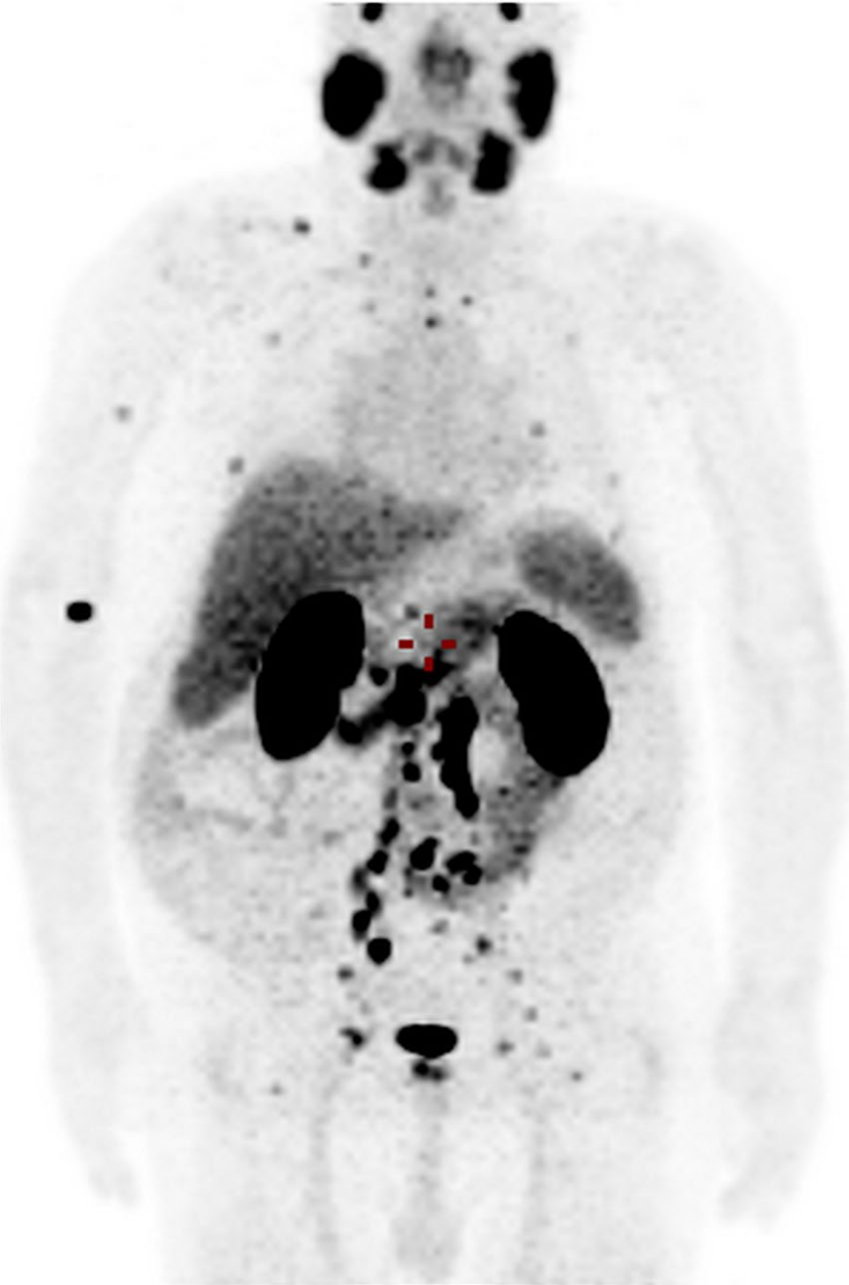
68Ga-isoPROtrace-11 maximum intensity projection PET image of a 76 years old patient diagnosed with prostate cancer 15 years ago (GS 7), presented with biochemical failure. MIP (Maximal Intensity Projection) image shows multiple bone and lymph node metastases as well as the normal biodistribution of the tracer.

Tumour-to-background ratio was further analyzed by dividing the average tumour uptake measurements to the liver and mediastinal blood pool activities. Overall, a total of 100 malignant lesions were measured (48 prostate, 24 LN, 22 bone and 6 soft tissue lesions) and the following results were received: average tumour-to-liver ratio was 2.7 (0.5–27.5) and average tumour-to- mediastinal blood pool ratio of 13.54 (1.9–148.2). Distribution of the tracer in different organs and in prostate cancer lesions is shown in Fig. 4.

## Discussion

In this study, we have shown the detailed *in-vivo* distribution of 68Ga-PSMA-11 in normal tissues and tumour lesions, while produced with a room temperature cold kit methodology, which allows for faster and easier preparation.

Physiological biodistribution of 68Ga-PSMA-11 produced with synthesis module was analyzed previously^16,17^ and the most recent studies using the same uptake parameter of SUVmean in the same organs were chosen for comparison. Dermici *et al*.^16^ analyzed normal distribution pattern of 68Ga-PSMA-11 in 56 disease-free patients and showed an average SUVmean of 1.7 in normal prostate gland. Afshar-Oromieh *et al*.^17^ analyzed 68Ga-PSMA-11 uptake both in normal tissues and tumour lesions in 37 patients and showed an average SUVmean of 2.9 in normal prostate. These results are similar to the results of the current study with an average SUVmean of 2.4 in normal prostate gland. Both studies showed similar biodistribution pattern with the highest uptake in kidneys, lacrimal and parotid glands. More detailed comparison is presented in Fig. 2. This similarity in uptake pattern implies that physiological biodistribution of 68Ga-isoPROtrace-11 is acceptable and thus may be used for imaging purposes.

The major limitations of this study are related to the assessment of pathological findings: patient population was very heterogeneous without inclusion criteria based on clinical indication, Gleason score or PSA levels. The exact effect of the irradiation or hormone therapy on the normal expression of PSMA in prostate gland and metastatic lesions was not taken into consideration as well, leading to a very big range of tracer uptake in pathologic lesions. In addition, histopathological confirmation of lesions was lacking due to the absence of long enough follow up period. Hence, our results in pathological group of findings required more detailed selection of patients to be compared to other published data, which was not in the scope of the current study. However, average SUVmax in most of the findings was 11 or more, with the only exception of LN metastases that showed relatively low SUVmax of 4.7 in one patient. Overall, the pathological findings (including prostate gland and metastatic lesions) had an excellent contrast demonstrating high average SUVmax of 13.9 (3.5–77) and the areas considered as background showed low SUVmean (ex., mediastinal blood pool had average SUVmean of 1.3 ± 0.4). Based on these findings, a cut-off value of SUVmax of 3.5 seems to be useful in our patient cohort for differentiating prostate tumours from normal prostate tissue, but due to the study’s limitations has to be proven in further research.

Lymph node metastases, as the most common metastatic site, showed high tracer uptake with average SUVmax of 14.6, ranging from 3.7 to 59.3. This high uptake resulted in an excellent target-to-non-target ratio allowing the detection of lymph nodes smaller than 15 mm in diameter. Moreover, in our cohort of patients, the biggest group of lymph nodes with pathological uptake was Group A (small LN up to 15 mm) which included 19 of all 22 patients suspected of having LN metastases. These results demonstrate that in randomly chosen cohort of patients, pathological PSMA uptake may be expected in small lymph nodes and it should be taken into consideration when reporting the scans.

Skeleton is another common site of prostate cancer metastasis and also showed high tracer uptake with an average SUVmax of 15.9, ranging from 4.3 to 77. That makes bone metastases to be easily detectable as well as lymph node metastases. It should be noted that in this group of patients most of the pathological findings were reported in Group D, which was the biggest and represented pathological PSMA foci with correspondent CT findings. Two patients, however, had pathological PSMA uptake and no correlative finding on CT (Group E). This finding may represent bone marrow metastases without reactive bone formation which is missed by CT and will, perhaps, be missed by bone scan as well. Bone scan and CT are the current standard of care which, when applied in these cases, will lead to an underestimation of the burden of disease with implication on therapy planning. This pattern can also be seen in a benign lesion or may be related to an artifact. These aspects should be taken into consideration while reporting the scans, making PSMA uptake alone (without a CT finding) an equivocal finding, requiring further investigation or follow up. Although our results show clinical applicability of 68Ga-isoPROtrace-11 in patients with prostate cancer, it should be noted that comparison to other available kits, using the same (ANMI) or a different (THP) molecule as isoPROtrace-11 was not performed as it is not in the scope of the current study. To the best of our knowledge, there is no technical advantage comparing to THP kit. The advantage of isoPROtrace-11 in regard to ANMI kit, which is demonstrated in a video provided by ANMI, is that the radiolabeling process consists of 3 vials and special connectors, as opposed to isoPROtrace-11, which is a 1 vial approach kit. A 1 vial approach provides a simpler for Ga68 labeling. Overall, using isoPROtrace-11 is a time-saving technique resulting in increased radiochemical yields of >98% and purity of >91% after 5 min.

Comparing to the mentioned advantages, a module-based synthesis requires a more professional operator to handle the system. In addition to the higher cost of instruments and consumables (module, pumps, reactors, etc.), a module-based synthesis may present with more room for errors due to the complexity of module operation.

A larger prospective study comparing all available kits for 68Ga-PSMA-11 production with a novel 1-step cold kit technology may be of value.

## Conclusions

This study provides 68Ga-isoPROtrace-11 biodistribution data in normal tissues and pathological findings, in a large patient subset, showing clinical applicability of the tracer produced with the cold kit technology. This approach may enable a robust, high quality, easy and quick labeling process.
